# SDF-1 involvement in orthodontic tooth movement after tooth extraction

**DOI:** 10.1038/s41598-024-55632-2

**Published:** 2024-02-29

**Authors:** Duangtawan Rintanalert, Yuji Ishida, Albert Chun-shuo Huang, Kasumi Hatano-sato, Kai Li, Pintu-on Chantarawaratit, Risa Usumi-fujita, Jun Hosomichi, Takashi Ono

**Affiliations:** 1 Department of Orthodontic Science, Graduate School of Medical and Dental Sciences, Tokyo Medical and Dental University (TMDU), Yushima 1-5-45, Bunkyo-ku, Tokyo, 113-8510 Japan; 2 Department of Orthodontics, Faculty of Dentistry, Chulalongkorn University, Bangkok, 10330 Thailand

**Keywords:** Orthodontic tooth movement (OTM), Stromal cell-derived factor 1 (SDF-1), Tooth extraction, Regional acceleratory phenomenon (RAP), Neutralizing antibody, Biomarkers, Orthodontics, Cell biology

## Abstract

The stromal cell-derived factor 1 (SDF-1)/chemokine receptor type 4 (CXCR4) axis plays a key role in alveolar bone metabolism during orthodontic tooth movement (OTM). Herein, the effects of the SDF-1/CXCR4 axis on the regional acceleratory phenomenon (RAP) in OTM velocity and on changes in the surrounding periodontium after adjacent tooth extraction in rats were investigated. Six-week-old male Wistar/ST rats underwent left maxillary first molar (M1) extraction and mesial OTM of the left maxillary second molar (M2) with a 10-g force closed-coil spring. Phosphate-buffered saline, immunoglobulin G (IgG) isotype control antibody, or anti-SDF-1 neutralizing monoclonal antibody were injected at the M1 and M2 interproximal areas (10 μg/0.1 mL) for the first three days. Analyses were performed after 1, 3, and 7 days (*n* = 7). The results demonstrated a significant increase in SDF-1 expression from day 1, which was effectively blocked via anti-SDF-1 neutralizing monoclonal antibody injection. On day 3, the M2 OTM distance and the number of positively stained osteoclasts significantly reduced alongside a reduction in inflammatory markers in the experimental group. Our results demonstrated that serial local injection of the anti-SDF-1 neutralizing monoclonal antibody reduces M2 OTM, osteoclast accumulation, and localized inflammatory responses in an OTM model with tooth extraction-induced RAP.

## Introduction

It has been well established that teeth adjacent to the extraction site can move more effectively for a certain amount of time than in usual non-surgical conditions^1,2^. Injuries such as bone fractures or tooth extraction can elicit a cascade of tissue reorganization activities and physiological healing events that occur in the surrounding tissues, leading to an acceleration of the normal ongoing tissue processes of both soft and hard tissues. This is known as the regional acceleratory phenomenon (RAP)^3^. The effects of the RAP on tooth movement have been reported in several studies that used minimally invasive surgery (MIS)^4–6^. However, additional surgery is required, which can raise concerns regarding its necessity. Despite its minimal invasiveness, surgery can lead to undesired pain, possible root damage, and a potential risk of infection and resorption of the alveolar bone^4,7,8^. Unlike in MIS, tooth extraction is usually unavoidable if planned. Because this is a compulsory procedure, patients are more likely to accept and undergo the treatment. Therefore, a better understanding of the mechanism underlying the RAP induced by tooth extraction during orthodontic tooth movement (OTM) is beneficial for maximizing treatment efficiency.

Stromal cell-derived factor 1 (SDF-1) is a well-known chemokine critical for the retention and migration of stem and progenitor cells^9,10^. Through interactions with its cognate receptor, chemokine receptor type 4 (CXCR4), SDF-1 is involved in the development of various organs and pathological conditions, including bone fractures^9,11,12^. Upregulation of this chemotactic factor in damaged tissues is well documented, along with its relationship with the reduction in oxygen or hypoxia found at the site of local injury^13^. An increase in SDF-1 expression, sequential inflammation, and local ischemia attracts CXCR4 + hematopoietic and mesenchymal stem cells with high differentiation and proliferation potential to the injured site to initiate the bone-healing process^9,14^. In relation to bone remodeling, researchers have found multiple valuable roles for the SDF-1/CXCR4 axis in regulating both osteoclasts and osteoblasts. This chemoattractant cytokine indirectly affects bone formation by promoting the recruitment of circulating osteoblast progenitor cells to areas of bone healing^13^. On the osteoclastic side, Wright et al^15^. concluded that, firstly, SDF-1 is a chemoattractant for osteoclast precursors; second, SDF-1 stimulates early osteoclastogenesis; and, third, SDF-1 can promote the survival of mature human osteoclasts^15^. In summary, SDF-1 is a possible key factor of bone remodeling associated with bone marrow stem cells, osteoblasts, and osteoclasts under both normal homeostatic and pathological conditions.

Recently, the SDF-1/CXCR4 axis was found to be closely associated with OTM. The experimental group that was administered the CXCR4 antagonist AMD3100 showed decreased OTM and osteoclast accumulation in rat molars under mechanical force application^16^*.* A similar study investigating the effects of local versus systemic serial administration of AMD3100 also reported less OTM and fewer osteoclasts accumulated on the periodontal ligament (PDL) compression side in both groups than in controls. Additionally, a reduction in both SDF-1 and CXCR-4 gene expressions was found alongside increased trabecular thickness in the AMD3100-treated groups, suggesting the effectiveness of AMD3100 in controlling alveolar bone metabolism during OTM^17^. Therefore, with this suggested the involvement of the SDF-1/CXCR4 axis in alveolar bone metabolism during OTM and successful inhibition using a receptor antagonist in this model, it can be concluded that SDF-1 and its CXCR4 receptor play a vital role in the bone remodeling process during OTM.

The role of SDF-1 in the RAP of the periodontal tissue is expected; however, its association has not yet been elucidated. Consequently, offering presently inadequate data and being able to elucidate and clarify this expected but unclear relationship would benefit the control of tooth movement in cases where tooth extraction is needed. This study aimed to evaluate the effects of the SDF-1/CXCR4 axis on the RAP in the OTM of rats after tooth extraction. Considering the relationship between SDF-1 and the RAP, we hypothesized that OTM of the maxillary second molar (M2) following maxillary first molar (M1) tooth extraction significantly increases the expression level of the SDF-1 chemokine in the SDF-1/CXCL4 axis, and blocking SDF-1 using an anti-SDF-1 neutralizing monoclonal antibody results in a significant reduction in OTM and significant bone morphological changes, histological changes, and gene expression levels compared to that in the control.

## Material and methods

Identification of the expression of SDF-1 in this animal model was analyzed alongside the SDF-1 and RAP relationship in this model through the localized injection of anti-SDF-1 neutralizing antibody, therefore, divided the analysis methods into two distinctive parts. All animal experiments were approved by the Institutional Animal Care and Use Committee of the Tokyo Medical and Dental University (A2021-115C) and were performed in accordance with relevant guidelines and regulations. Our study is reported in accordance with ARRIVE guidelines.

### Animals

Six-week-old male Wistar/ST rats, shipped at 5-week-old, with a mean weight of 180 g, were used in the study. All animals were housed in the same room under controlled temperature, humidity, and light conditions. A standard alternating 12-h light/dark environment was maintained. All animals were given a week for acclimatization, a powdered diet (RI-Sterile feed CE-2 powder type; CLEA Japan, Tokyo, Japan), and water ad libitum. The powdered food diet was replaced on the day of the experiment (tooth extraction, OTM, and medicine injection) to reduce trauma to the soft tissue after tooth extraction, ease pain, and reduce the risk of orthodontic appliance damage. The health status and body weight of the rats were evaluated on the first three days after the surgical procedures and every other day throughout the experiment. All procedures were performed under general anesthesia.

### Tooth extraction and orthodontic tooth movement

Sixty-three rats were randomly allocated to three groups of 21 rats each.

1. PBS Control group: M1 tooth extraction, M2 OTM, PBS injection
2. IgG Control group: M1 tooth extraction, M2 OTM, IgG isotype control antibody injection (10 μg/0.1 mL)
3. SDF-1 Antibody group: M1 tooth extraction, M2 OTM, anti-SDF-1 neutralizing monoclonal antibody injection (10 μg/0.1 mL)

All treatment procedures were performed following a subcutaneous injection of three-mixed anesthesia (medetomidine, 0.3 mg/kg; midazolam, 4 mg/kg; butorphanol, 5 mg/kg). The rats were weighed to calculate the anesthetic drug dose prior to each injection. To generate the RAP response, the left maxillary first molar was gently elevated and extracted with the least possible damage to the adjacent second molar. Subsequently, OTM was initiated using a 10-g force nickel-titanium alloy closed-coil tension spring (Tomy International, Tokyo, Japan) attached to the cervical ligature wire loop (Tomy International; diameter: 0.20 mm) around the left maxillary second molar to the ligature wire loop at the most cervical portion of the upper incisors and fixed with light-cured composite resin (GC, Tokyo, Japan). This generated a constant 10 g mesial force after activation, as measured by a force gauge (Supplementary Fig. 1a–c).

After the coil spring placement, the experimental group was given a local injection of anti-SDF-1 neutralizing monoclonal antibody (MAB310; R&D Systems, Minneapolis, MN, USA) at a volume and concentration of 10 μg/0.1 mL. Injections were administered in the buccal and palatal gingival mucosa of the interproximal area between the left maxillary first and second molar, 0.05 mL on each side. The control group received either a PBS injection or IgG isotype control antibody (MAB002; R&D Systems) injection at the same volume, concentration, and injection site as the experimental group. The contralateral side of the maxilla was not treated in all groups. All rats were treated with penicillin (Benzathine G penicillin, 20,000 IU) and painkiller (Buprenorphine, 0.01 mg/mL) intramuscularly after the surgical procedures. Medetomidine antagonists were administered for recovery from general anesthesia.

Appliances were routinely checked and cleaned on the first three days and every other day to prevent any possible mechanical damage to the coil spring and loosening of the wire ligatures.

Seven rats from each group were sacrificed 1, 3, and 7 days after the surgical procedures, according to their timeline. Serum samples were collected from all rats to quantify the SDF-1 protein concentration in the peripheral blood using an ELISA (*n* = 7). Tissues were evaluated for bone morphology, histological changes (*n* = 3), and molecular biology by quantitative reverse transcription PCR (RT-qPCR) (*n* = 4) (Supplementary Fig. 1d).

With the objective of identifying SDF-1 expression in this model, we observed the relationship between SDF-1 and the RAP via the localized injection of an anti-SDF-1 neutralizing antibody. The analyses of the obtained results were separated into two parts.

**Part 1.** To test the SDF-1expression, the untreated control side and the M1 extraction and M2 OTM experimental side of the control PBS injection group were used. Using all three time point subgroups, the collected maxillae were halved, and the two sides within the same group were compared. Analysis of the presence and location of SDF-1 was performed by IF staining. In addition, PCR analysis was performed to confirm the mRNA expressions of SDF-1 and its receptor CXCR4 at the transcriptional level.

**Part 2.** To assess the effects of SDF-1 blockade using a neutralizing antibody on the extraction of M1 and OTM in the M2 model, all groups and subgroups were compared using only their experimental sides. Micro-computed tomography (micro-CT) analysis of tooth movement and bone morphology, ELISA of blood serum to test the systemic effects of the medicine, histological analysis using hematoxylin and eosin (H&E) staining, histochemical staining for TRAP, IHC staining, IF staining, and RT-qPCR were performed to quantify gene expression levels.

### Micro-CT analysis

In vivo micro-CT (R_mCT2 SPMD; Rigaku, Tokyo, Japan) of the rat head was performed both before and immediately after the surgical procedures for all rats. Preoperative micro-CT data were used as a baseline for M2 tooth movement measurements, and post-operative micro-CT data were used to check for broken root remnants of M1 immediately after tooth extraction. During the active tooth movement period, micro-CT imaging was performed under general anesthesia on days 1, 3, and 7 of the experimental timeline for each group. All imaging conditions were set at 90 kV, 160 mA, and a 30 mm field of view. The tooth movement distance of M2 was measured from the micro-CT images using two methods.

1. Distance from the distal surface of M2 to the mesial surface of maxillary third molar (M3).
2. The measurement from the reference line was created from the opposite M3 of the maxillary right untreated side to the distal surface of M2.

The mesial movement of M3 was also analyzed using the second method (Supplementary Fig. 2a–c).

Three rats from each subgroup were randomly selected after sacrifice for histological and bone morphological analyses. Dissected maxillae were immediately fixed by immersing in 4% paraformaldehyde (pH 7.4, FUJIFILM Wako Pure Chemical Corporation, Osaka, Japan) at 4 °C for 48 h and stored in PBS pH 7.4 at 4 °C. Samples were scanned using a micro-CT system coupled to a desktop X-ray micro-CT system (SMX-100CT; Shimadzu, Kyoto, Japan) with output settings of 75 kV and 140 mA, and a scanning resolution of 9.5 μm. The ROI for structural morphometry analysis was the interradicular alveolar bone of the maxillary M2, defined by the borders of the septum between the roots of the M2. Using trabecular bone analysis software (TRI/3D-BON; Ratoc, Tokyo, Japan), bone parameters regarding BV/TV, BMD, Tb.Th, Tb.N, and Tb.Sp were analyzed in the selected ROI. Evaluations of the micro-CT data were repeated at least three times by a single examiner to calculate the average values (Supplementary Fig. 2d,e).

### Enzyme-linked immunosorbent assay

Serum SDF-1 protein levels were determined using an ELISA kit (MCX120; R&D Systems), following the manufacturer’s protocol. Under general anesthesia, 5 mL of blood was drawn via cardiac puncture at the time of sacrifice. Serums were separately collected using a serum separator blood collecting tube (BD Vacutainer® SST™ II Plus; BD, Franklin Lakes, NJ, USA), centrifuged at 1000 g for 10 min, and stored at − 80 °C until analysis. The ELISA was performed in duplicate for each sample. The colorimetric density of the developed microplate was determined using a Multiskan Sky Microplate Spectrophotometer and SkanIt software (Thermo Fisher Scientific, Waltham, MA, USA) at 450 nm. Wavelength corrections were performed by subtracting the readings at 540 nm or 570 nm from those at 450 nm for optical imperfection correction.

### Hematoxylin and eosin staining

Following micro-CT imaging, the specimens were decalcified in 10% disodium ethylenediamine tetraacetate in phosphate buffer at pH 7.4 for six weeks, dehydrated in a series of ascending ethanol concentrations, cleared with xylene, infiltrated, and embedded in paraffin. A rotary microtome was used to obtain 4 μm-thick serial sections in a horizontal plane through the first, second, and third-molar roots. The sections were mounted on coated glass slides, deparaffinized with xylene, and stained with H&E. Morphological and histological changes were examined by light microscopy (DXm1200; Nikon, Tokyo, Japan) using NIS-Elements D Imaging Software (Version 2.30, Nikon).

### Immunofluorescence staining

The expression of SDF-1 and its presence and location were evaluated by IF staining using rabbit anti-SDF-1/CXCL12 polyclonal antibody (Bioss, Woburn, MA, USA). Deparaffinized and rehydrated sections were blocked with 5% bovine serum albumin for 60 min at room temperature (25 °C). Sections were incubated with primary antibody (rabbit anti-SDF-1/CXCL12 polyclonal antibody; Bioss) (1:100 dilution) overnight at 4 °C. Secondary antibodies (Donkey Anti-Rabbit IgG H&L Alexa Fluor® 594; Abcam, Cambridge, UK) (1:200 dilution) were used to incubate the sections for 40 min in room temperature. Quenching of autofluorescence (Vector TrueVIEW; Vector Labs, Burlingame, CA, USA) was performed to remove unwanted fluorescence in the tissue sections following the manufacturer’s protocol. Sections were mounted with VECTASHIELD Vibrance Antifade Mounting Medium with DAPI (Vector Labs, Burlingame, CA, USA), as provided in the kit. Fluorescent images were evaluated within 48 h of mounting using a BZ-X710 fluorescence microscope (Keyence, Itasca, IL, USA).

### Tartrate-resistant acid phosphatase staining

To analyze catabolic activity in the alveolar bone, multinucleated osteoclasts and preosteoclasts were stained with tartrate-resistant acid phosphatase (TRAP). After deparaffinization and rehydration, the sections were stained using a TRAP staining kit (Wako Pure Chemical, Osaka, Japan) according to the manufacturer’s protocol. Incubation of the sections in TRAP buffer for 30 min was performed, followed by washing with distilled water, and counterstained with 0.02% Fast Green (Wako Pure Chemical, Osaka, Japan) nuclear staining solution for 10 min. The number of TRAP-positive multinucleated osteoclasts in the PDL area around the M2 mesial roots was counted by a single examiner in three randomized sections for each sample and the averages were calculated.

### Immunohistochemistry staining

The sections were stained with the following primary antibodies for the IHC analyses: IL-1β, IL-6, RANKL, and Cathepsin K (Bioss, Woburn, MA, USA) (1:400 dilution). After deparaffinization and rehydration, sections were treated with 3% hydrogen peroxide (Abcam) for 10 min to quench the endogenous peroxidase activity, followed by the incubation with normal goat serum to block the non-specific binding for 30 min at room temperature. Primary antibodies with different specific concentrations were applied to the sections and incubated overnight at 4 °C. The following day, the slides were incubated with the biotinylated secondary antibody for 30 min using the VECTASTAIN Elite ABC Rabbit IgG Kit (Vector Labs, Burlingame, CA, USA). Subsequently, the prepared VECTASTAIN ABC reagent was applied to the slides and incubated for another 30 min. Sections were stained with 3,3’-Diaminobenzidine (DAB) (Abcam) and counterstained with hematoxylin.

### Quantitative reverse transcription PCR

Four rats from each subgroup were randomly selected for the quantification of gene expression using RT-qPCR. Immediately after sacrifice, the maxillae were dissected from the skulls and any attached tissues were removed. The maxilla was trimmed with sterile scissors and the M2 clinical crown was removed using a sterile orthodontic wire cutter. The attached gingival tissue was discarded, leaving the final sample consisting only of the M1 extraction socket, M2 roots with the adjacent PDL attached, and the surrounding bone at a distance of approximately 1–2 mm. For the untreated negative control, tissue samples were collected as described in Part 1. The M1 crown was removed along with the M2 crown and the surrounding tissue sample size was identical to that previously stated. Tissue samples were transferred to liquid nitrogen and snap-frozen immediately after collection. A sterile mortar and pestle with liquid nitrogen was used to grind the collected samples into small pieces. The crushed samples were further homogenized, and total RNA was isolated using a TRIzol® reagent (Invitrogen; Thermo Fisher Scientific). Complimentary DNA was synthesized from total RNA with reverse transcription using PrimeScript™ RT Master Mix (Takara Bio, Shiga, Japan) according to the manufacturer’s instructions. Real-time PCR analysis was done in triplicate for each sample using Probe qPCR Mix (Takara Bio) and commercially obtained rat’s specific primers for CXCL12/SDF-1, CXCR4, IL-1β, RANKL, OPG and GAPDH (TaqMan Gene Expression Assay, Applied Biosystems; Thermo Fisher Scientific). Gene expression levels were calculated using the comparative Ct method and normalized to those of GAPDH.

## Statistical analyses

All measurements were repeated three times, and intraobserver reliability was determined using the intraclass correlation coefficient. The statistical analysis program was used for all tests. Normality tests were performed using the Shapiro–Wilk test to examine the data distribution for every set of data.

For results obtained in Part 1 in the Materials and Methods section, the untreated control side and experimental side were compared only in the control PBS injection group, and data with normal distributions underwent parametric comparisons with the unpaired *t*-test. If the data distribution was not normal, nonparametric comparisons were performed using the Wilcoxon signed-rank test for matched pairs.

The results obtained in Part 2 in the Materials and Methods section, the experimental side from all groups and subgroups were compared, and data with normal distribution underwent parametric comparisons with analysis of variance, followed by post-hoc analysis. If the data distribution was not normal, nonparametric comparisons with the Kruskal–Wallis test were performed, followed by post-hoc analysis. The level of significance was set at *p* < 0.05.

## Results

### Part 1

#### Immunofluorescence staining

**Figure 1:**
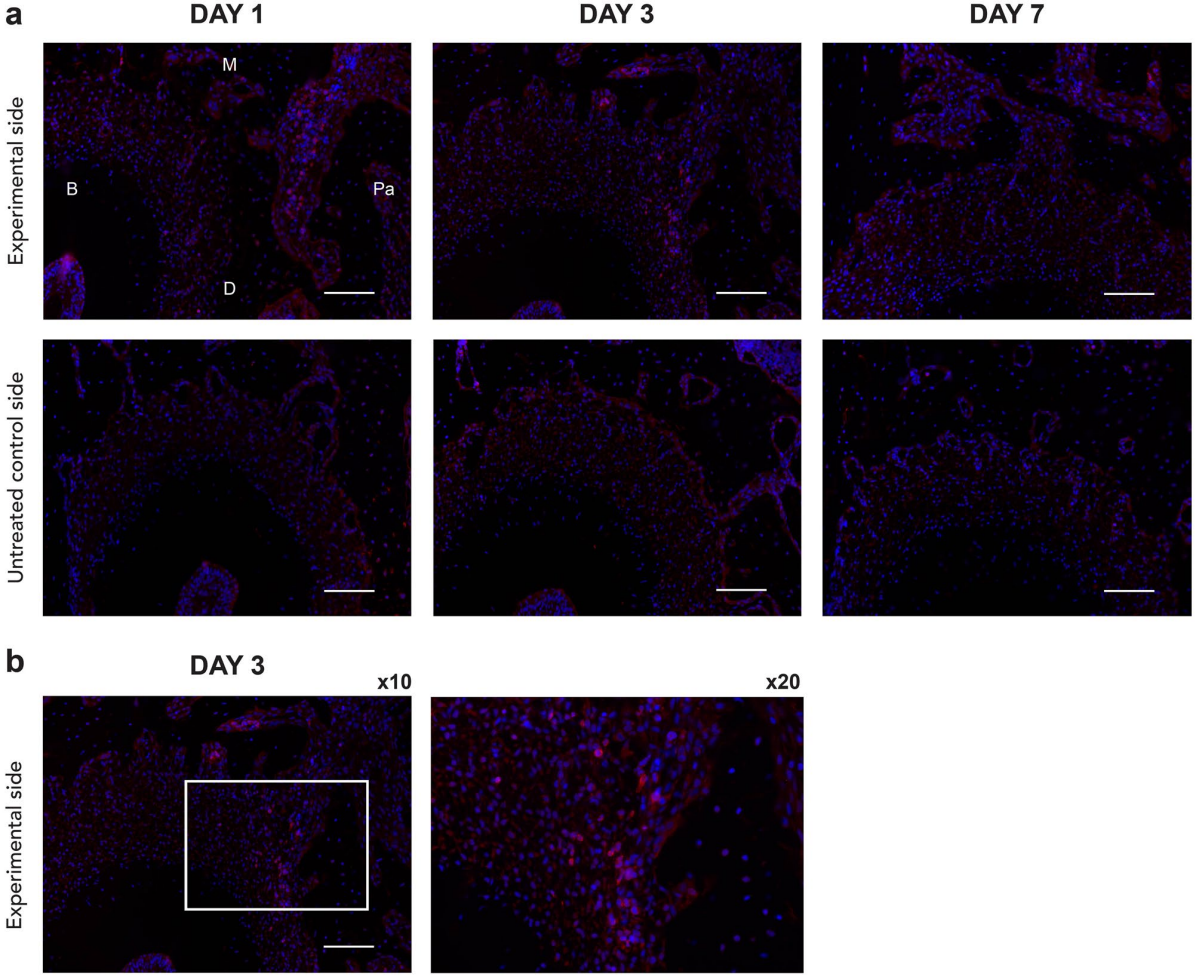
Immunofluorescent evidence of increased stromal cell-derived factor 1 (SDF-1) expression on the experimental side of the left maxillary second molar (M2) mesial root on days 1,3, and 7 (**a**). SDF-1 immunofluorescence (IF) staining of M2 mesial root at the compression side on day 3 of the experimental side at × 10 and × 20 magnification (**b**). Positively stained cells with Alexa Fluor® 594 are red, and the nuclei with DAPI are blue. Scale bars = 50 μm. Abbreviations: B, buccal; M, mesial; Pa, palatal; D, distal.

To identify the presence and location of SDF-1 expression in this experimental animal model, SDF-1 immunofluorescence (IF) staining of the M2 mesial root clearly revealed positively stained cells on the experimental side. Visualized by the bright, red-fluorescent dye of Alexa Fluor® 594, SDF-1 presence can be distinctively observed on days 1 and 3. Evident expression was detectable in the bone marrow area of the alveolar bone adjacent to the mesial root on day 1 and in the PDL of the compression side of the mesial root on day 3. SDF-1 expression was reduced on day 7, with some positive signals that could be traced around the PDL near the alveolar bone border and some in the bone marrow (Fig. 1a,b).

#### Quantitative reverse transcription PCR

**Figure 2:**
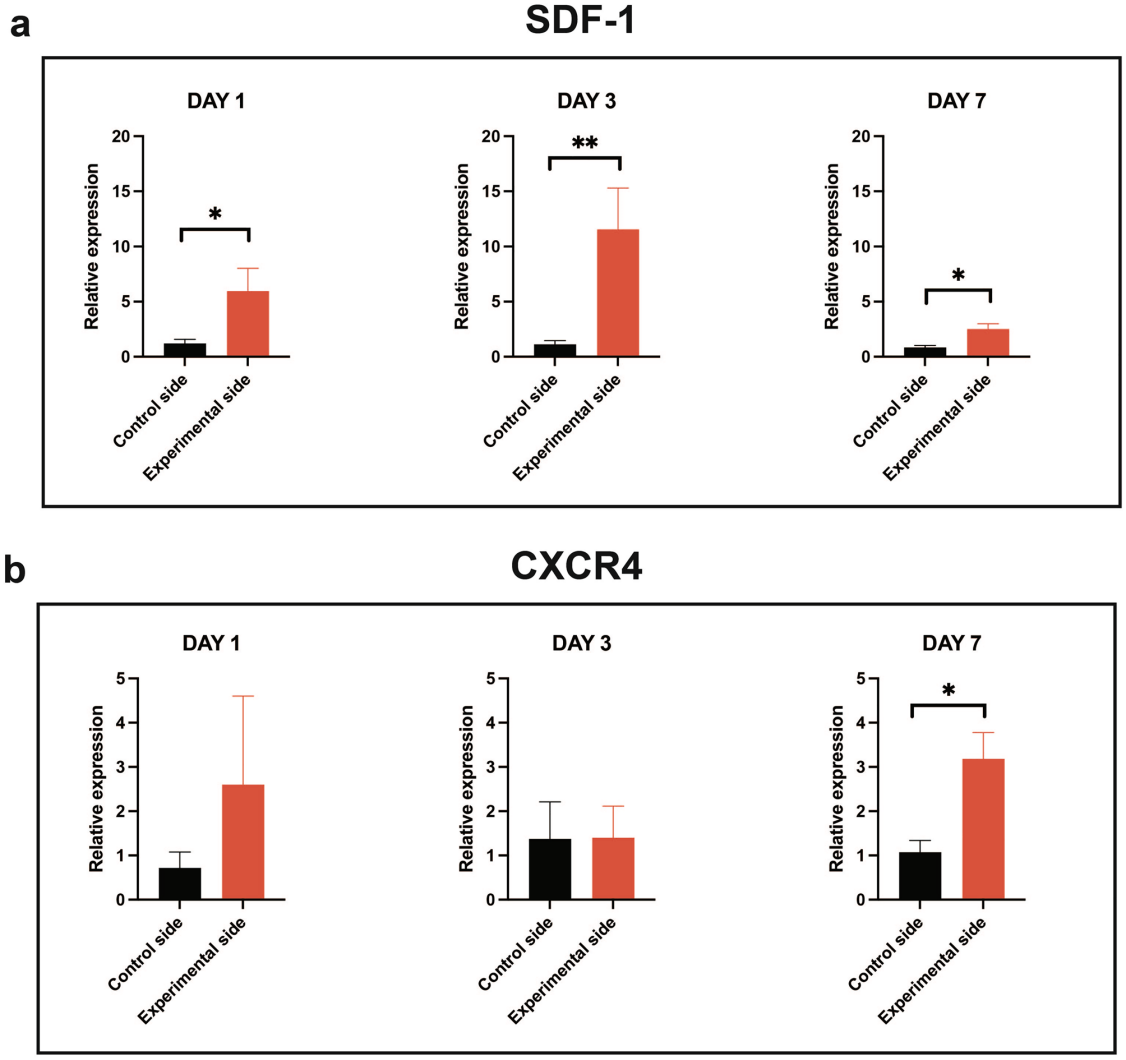
Qualitative reverse transcription polymerase chain reaction comparing the untreated control side and the experimental side of the control phosphate-buffered saline (PBS) group to verify the expression of our targeted chemokine (**a**) the stromal cell-derived factor 1 (SDF-1) and its receptor, (**b**) the chemokine receptor type 4 (CXCR4). A significant increase in the SDF-1 relative expression was detectable on days 1, 3, and 7 on the experimental side, whereas CXCR4 expression significantly increased only on day 7 in the experimental side. Values are presented as means ± standard deviation (*n* = 4). * *p* < 0.05, ** *p* < 0.01.

By testing the expression of SDF-1 during the extraction of M1 and OTM in the M2 model, the relative gene expressions of both SDF-1 and CXCR4 were found to be upregulated on the experimental side compared with that on the untreated control side. Significant differences were observed at all time points for SDF-1 gene expression, whereas a significant difference was observed only on day 7 for CXCR4 gene expression (Fig. 2a,b).

## Part 2

### Micro-CT analysis

#### Tooth movement

**Figure 3:**
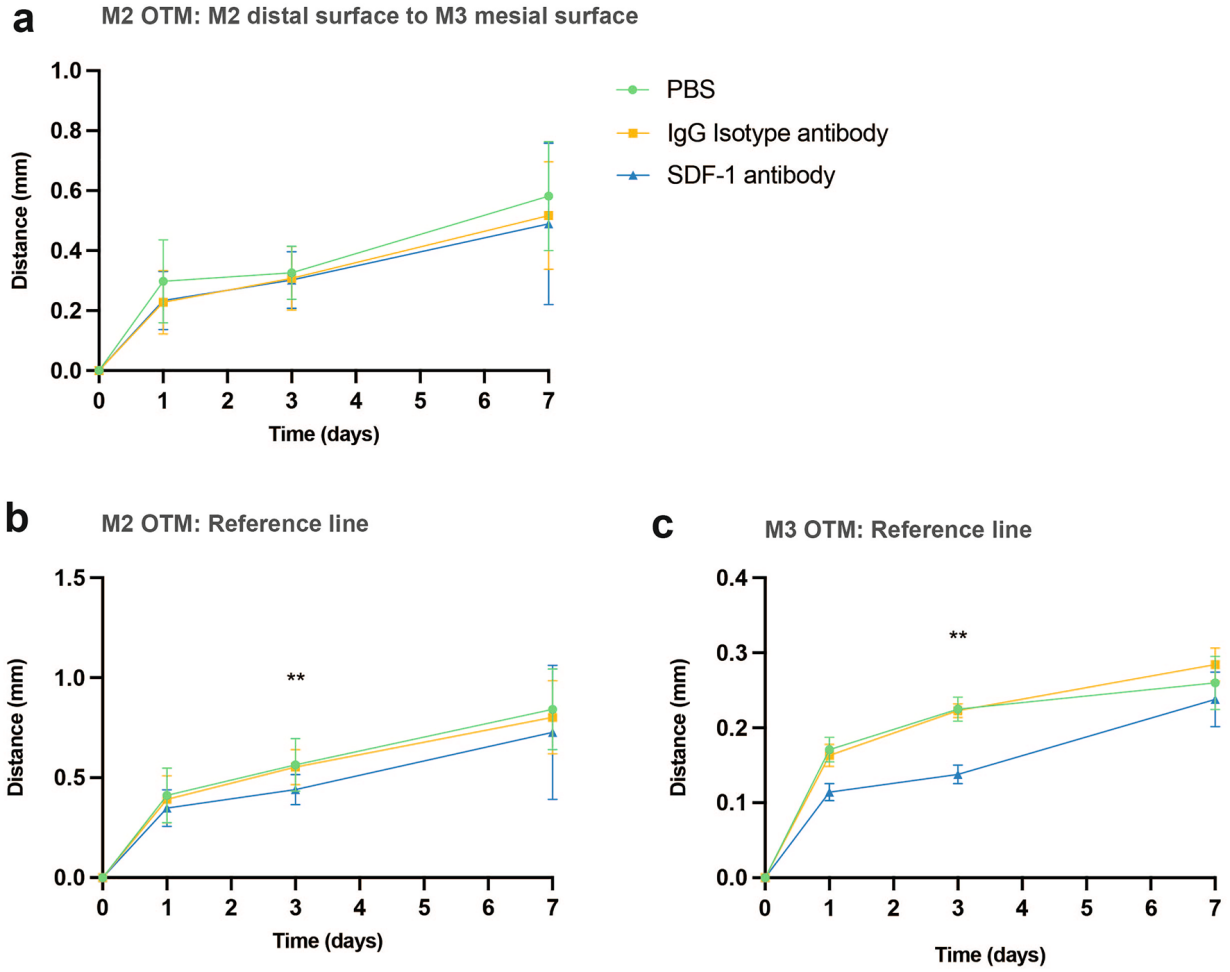
The maxillary second molar (M2) tooth movement distance after mechanical loading. Measured using the direct distance from M2 distal surface to maxillary third molar (M3) mesial surface, no significant differences were found in all groups at all time points (**a**). A measurement with the reference line created from the opposite M3 of the untreated side revealed a significant difference on day 3 of the experimental stromal cell-derived factor 1 (SDF-1) antibody group where less distance could be observed in comparison to the two control groups (**b**). Measurement of M3 tooth movement distance using the reference line created from the opposite M3 of the right maxillary untreated side. A significant difference was detectable on day 3 in the SDF-1 antibody group where less M3 tooth movement was measured in comparison to the two control groups (**c**). Values are presented as means ± standard deviation (*n* = 7). * *p* < 0.05, ** *p* < 0.01.

A significant difference was observed on day 3 after the initiation of OTM, measured using the reference line created from the opposite maxillary third molar (M3). At this time point, a shorter distance was observed in the SDF-1 antibody group than in the two control groups. Conversely, no significant differences were found in all groups at any time point when measured using the direct distance between the M2 distal surface and the M3 mesial surface (Fig. 3a,b). Therefore, further analysis was performed on the M3 mesial tooth movement using the opposite M3 reference line. In accordance with M2 tooth movement, a significant difference was also detected on day 3 in the SDF-1 antibody group, where less M3 tooth movement was measured in comparison with the two control groups (Fig. 3c).

#### Bone analyses

Among all parameters measured, only bone mineral density (BMD) was found to have a statistically significant difference on day 3 between the experimental SDF-1 antibody group and the two control groups. Treatment with localized anti-SDF-1 neutralizing monoclonal antibody injection resulted in higher BMD values than the two controls, in which either PBS or IgG isotype control antibody injections were administered locally. Notably, the graphs demonstrated a trend of reduction in bone volume fraction (BV/TV), trabecular thickness (Tb.Th), and trabecular spacing (Tb.Spac) in all groups during the experimental period from day 1–7, whereas trabecular number (Tb.N) showed an increasing pattern. This is in consideration of the differences in the mentioned parameters among the groups at each time point not being significant (Supplementary Fig. 3).

### Enzyme-linked immunosorbent assay

There was no significant difference in the SDF-1 protein concentration in the collected peripheral blood serum among all groups at any time point (Supplementary Fig. 4).

### Hematoxylin and eosin staining

In all groups, morphological examination of the mesial root of the M2 revealed narrowing of the PDL space on the compression side, along with the presence of Howship’s lacunae at the adjacent alveolar bone on that side. On the opposite side of the tension, widening of the PDL space and stretching of the collagen fibers was observed with finger-like projections of the alveolar bone.

### Immunofluorescence staining

**Figure 4:**
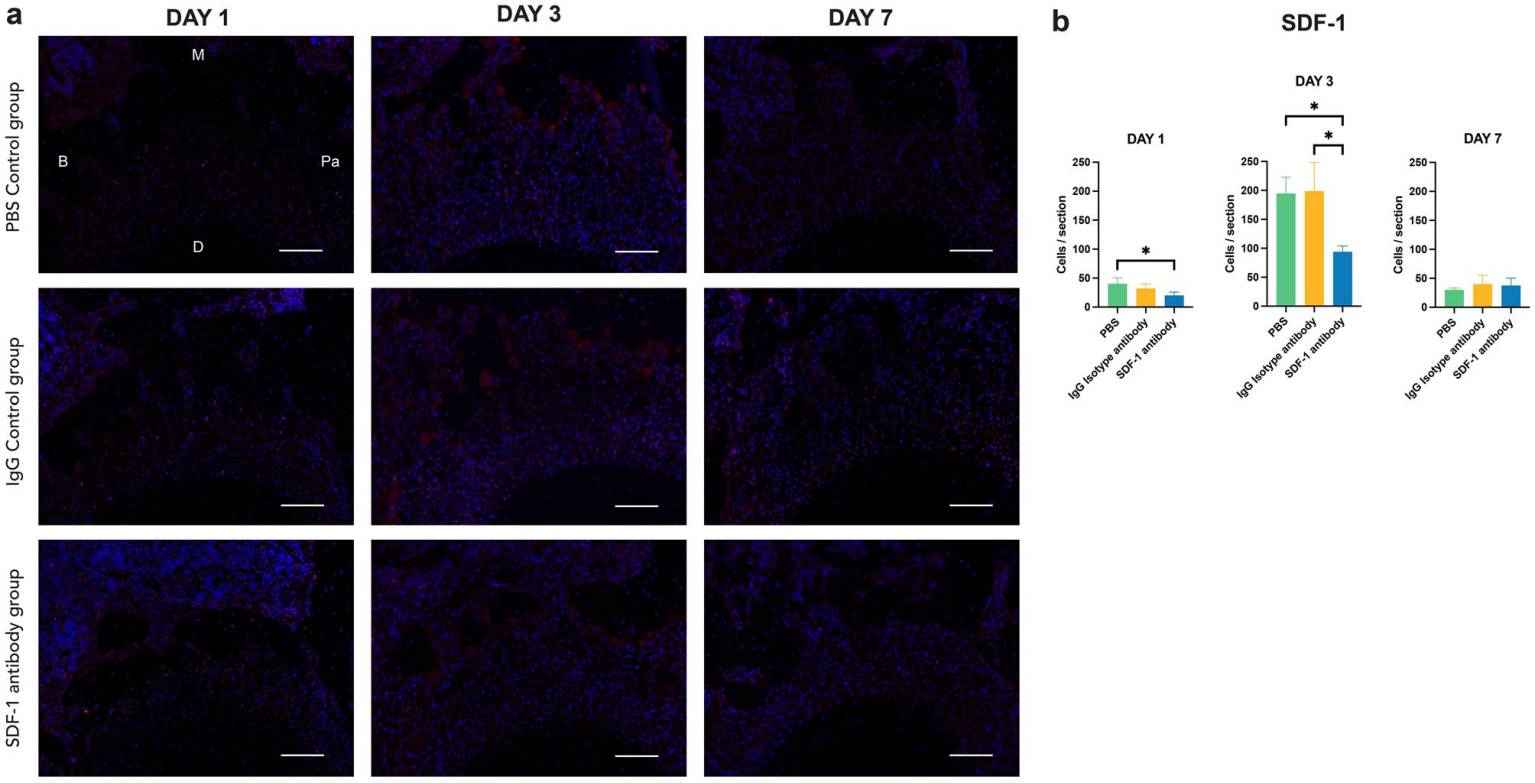
Immunofluorescence (IF) staining of stromal cell-derived factor 1 (SDF-1) using Alexa Fluor® 594 (red) and DAPI (blue) for nuclear staining at the compression side of maxillary second molar (M2) mesial root on days 1,3, and 7. Positively stained cells were detected abundantly on day 3, whereas less were observed on days 1 and 7. Following neutralization via anti-SDF-1 neutralizing monoclonal antibody injection, reduced positive signals could be detected on day 3 of the SDF-1 antibody group compared to the two control groups (**a**). Number of positively stained cells at the compression side of M2 mesial root on days 1, 3, and 7. Statistically significant differences can be seen on day 1 between the SDF-1 antibody group and the PBS control group, and on day 3 between the SDF-1 antibody group and the two control groups (**b**). Scale bars = 50 μm. Abbreviations: B; buccal, M; mesial, Pa; palatal, D; distal. Values are presented as means ± standard deviation (*n* = 3). * *p* < 0.05.

When defining the presence of SDF-1 after neutralization with the specific antibody, we found a statistically significant reduction in the positive emitting signals on day 3 and day 1 of the SDF-1 antibody group compared to that in the two controls and that in the PBS control group, respectively. No statistically significant differences were observed on day 7 between all groups or on day 1 between the IgG isotype antibody control group and the SDF-1 antibody group (Fig. 4a,b).

### Tartrate-resistant acid phosphatase staining

**Figure 5:**
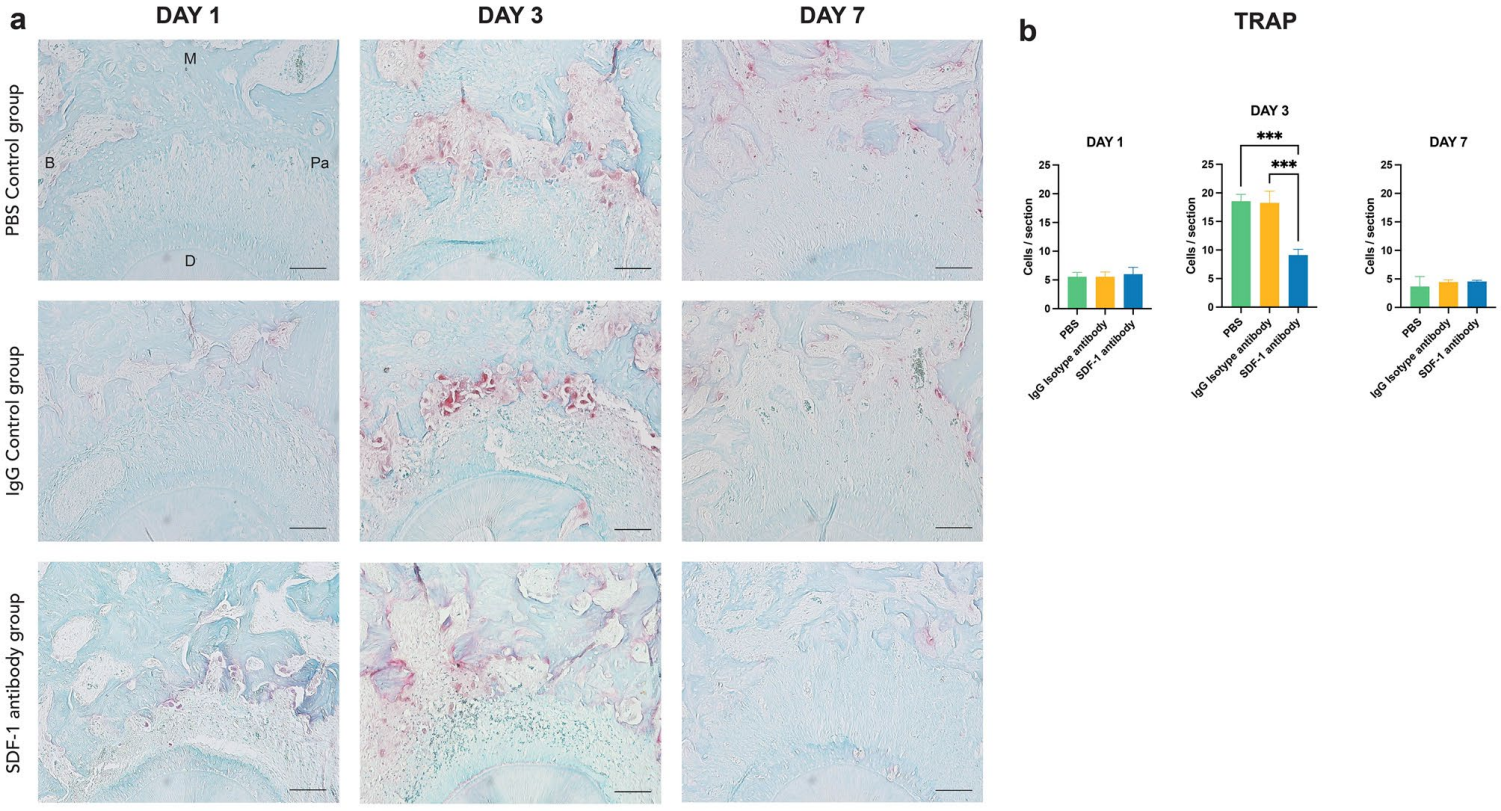
Representative image of tartrate-resistant acid phosphatase (TRAP) staining at the compression side of M2 mesial roots on day 1, 3, and 7. TRAP-positive multinucleated cells were observed abundantly on day 3 in both control groups, whereas less were observed on days 1 and 7 in all groups. Focusing on the compression side of the mesio-palatal root on day 3 of all three groups, the stromal cell-derived factor 1 (SDF-1) antibody group demonstrated less TRAP-positive multinuclear osteoclasts in the PDL compared to the two control groups (**a**). Number of TRAP-stained cells at the compression side of M2 mesial root on days 1, 3, and 7. Statistically significant differences can be seen on day 3 between the SDF-1 antibody group and the two control groups (**b**). Scale bars = 50 μm. Abbreviations: B; buccal, M; mesial, Pa; palatal, D; distal. Values are presented as mean ± standard deviation (*n* = 3). *** *p* < 0.001.

A marked increase in tartrate-resistant acid phosphatase (TRAP)-positive multinucleated osteoclasts was observed on the compression side of the M2 root on day 3 (Fig. 5a). At this point, during the entire experimental timeline, a statistically lower number of positively stained cells in the PDL of the M2 compression side was found in the SDF-1 antibody group compared to that in the two controls. Some positive cells were detectable on days 1 and 7 in all groups; however, the numbers were significantly lower than those on day 3, and no statistically significant differences were found among the three groups at these two time points (Fig. 5b).

### Immunohistochemistry staining

**Figure 6:**
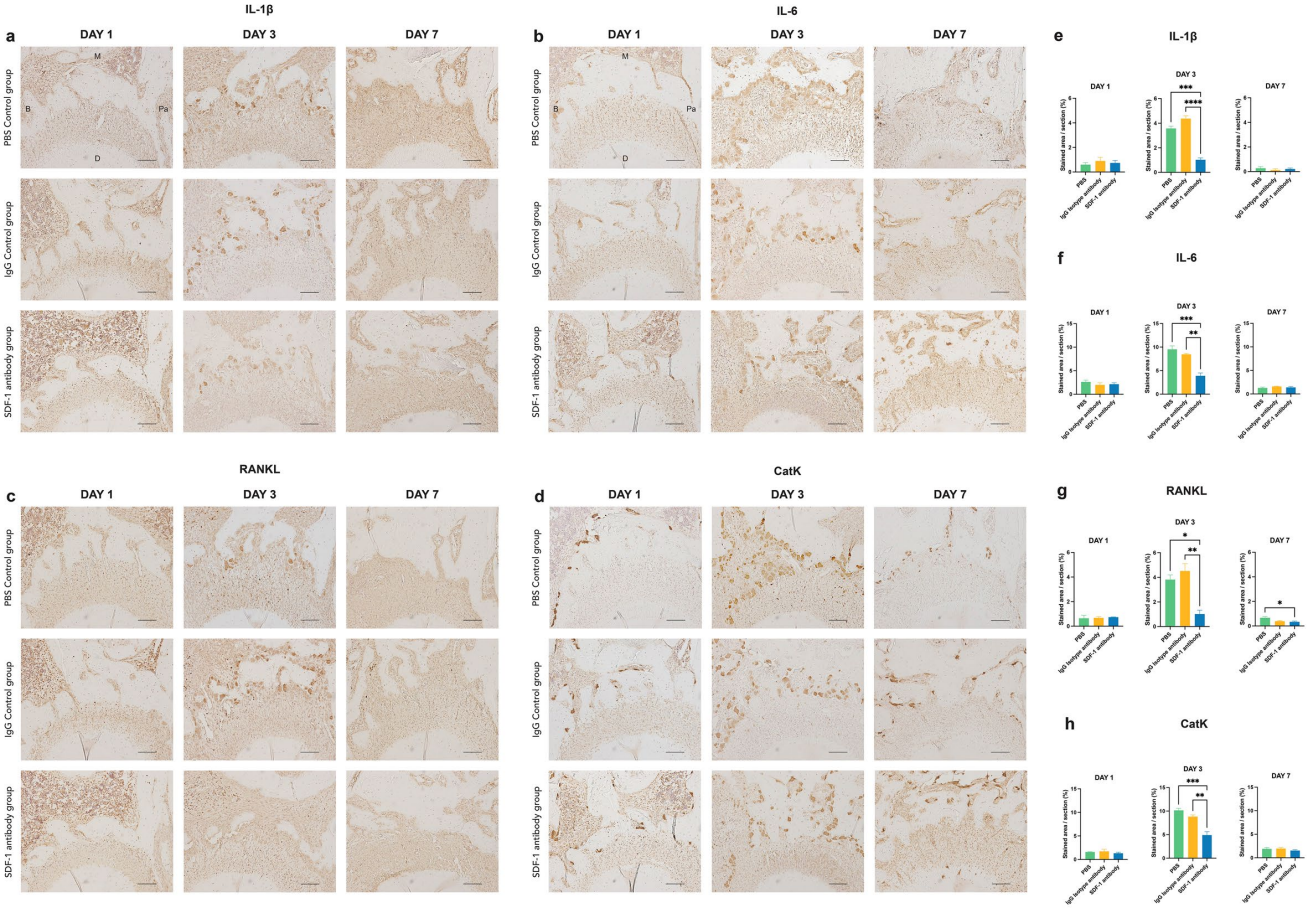
Immunohistochemistry-stained images of the M2 compression side, and quantitative analysis of positively stained areas of interleukin (IL)-1β) (**a**,**e**), IL-6 (**b**,**f**), receptor activator of nuclear factor-kappa B ligand (RANKL) (**c, g**), and Cathepsin K (CatK) (**d**,**h**). In all tested antibodies, significant reductions in positively stained areas per slide of the M2 compression side were observed on day 3 in the SDF-1 experimental group. * *p* < 0.05, ** *p* < 0.01, ** *p* < 0.001.

Statistically significant reductions in the percentage of the positively stained area per slide of the M2 compression side were found in the SDF-1 antibody group on day 3, compared to the controls when tested for interleukin (IL)-1β, IL-6, receptor activator of nuclear factor-kappa B ligand (RANKL), and Cathepsin K. No statistically significant difference can be seen on days 1 and 7 between all groups in the tested antibodies (Fig. 6a–h).

### Quantitative reverse transcription PCR

**Figure 7:**
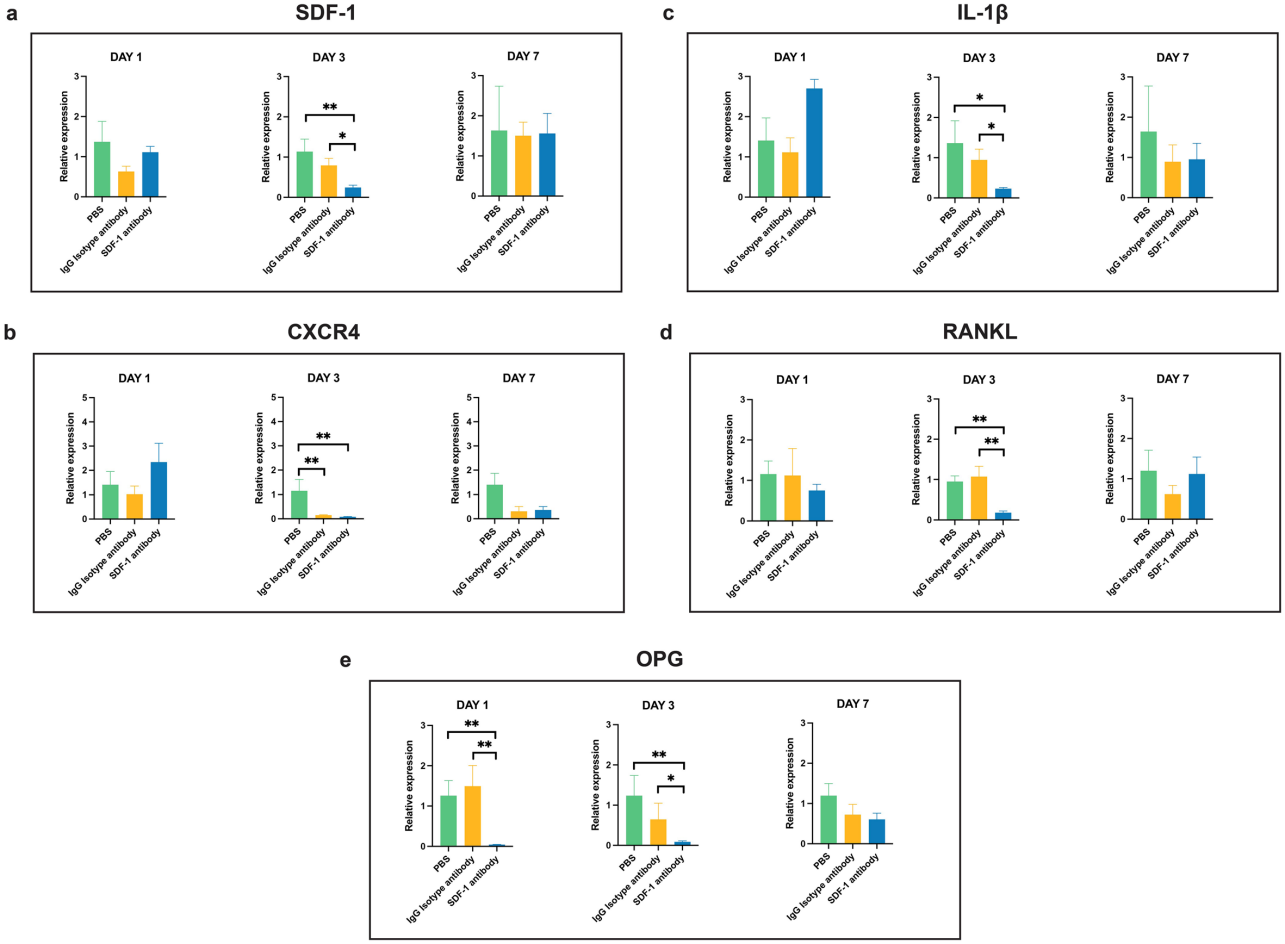
Qualitative reverse transcription polymerase chain reaction analyzing key genes involved in the inflammation, bone remodeling and stromal cell-derived factor 1 (SDF-1)/ chemokine receptor type 4 (CXCR4) axis related to the OTM. Relative gene expressions of SDF-1 (**a**), CXCR4 (**b**), interleukin-1 beta (IL-1β) (**c**), receptor activator of nuclear factor-kappa B ligand (RANKL) (**d**), and osteoprotegerin (OPG) (**e**) were analyzed using GAPDH as an internal reference. Expressions of all tested key genes were significantly reduced on day 3 in the experimental group, compared to the two controls. Additionally, a significant reduction in OPG was also found on day 1 in the experimental group. Values are presented as means ± standard deviation (*n* = 4). * *p* < 0.05, ** *p* < 0.01.

After assessing the effects of SDF-1 blockade, significantly downregulated mRNA expression levels were observed on day 3 in the experimental SDF-1 antibody group when tested for both SDF-1 and CXCR4. The IL-1β, RANKL, and osteoprotegerin (OPG) relative gene expressions were downregulated in the experimental group compared to that in the two control groups. Notably, a significantly decreased expression of OPG was observed on day 1 in the experimental SDF-1 antibody group compared to that in the PBS and IgG isotype antibody groups, without significant differences observed on day 1 in the other tested gene expressions. No significant differences were observed on day 7 in any of the selected relative gene expression levels (Fig. 7a–e).

## Discussion

Shortening the treatment time by accelerating tooth movement has become one of the main objectives for providing effective treatment within an optimized time. Surgical methods have long been used to improve this efficiency and elicit RAP, which is the amplification of inflammatory cytokine expression in response to physical trauma due to the natural inflammatory response of the body^5^. Although mild RAP activity can be triggered by orthodontic force application to periodontal tissues, the RAP can be maximized when tooth movement is combined with localized traumatic injury^3^. Initial acute inflammation and the host immune response trigger the recruitment and activation of stem and progenitor cells to migrate from a distant site to the injured site. This has been proposed as a compulsory element of osseous healing^10,18^. Although the cell origin involved in hard and soft tissue healing of the extraction socket remains unclear, studies have suggested that bone marrow-derived stem cells (BMSCs) could be the source of cells responsible for reparations^10,19^. The SDF-1/CXCR axis, which attracts CXCR4 + cells through a concentration gradient, is thought to be a master regulator of stem and progenitor cell migrations^9^ involved in the normal homeostatic regulation of bone remodeling, and this axis is possibly the key factor associated with BMSCs, osteoblasts, and osteoclasts^13,15^.

The upregulation of the SDF-1 chemotactic factor in damaged tissues has been well documented where irradiated bone marrow and fractured bone have presented an increase in SDF-1 expression, leading to chemotactic recruitment of CXCR4 + stem cells to the injured area^9,20,21^. In addition to the localized upregulation of inflammatory cytokines, which is usually expected in tissue injuries, a reduction in the level of oxygen or hypoxia is also observed in lesions owing to the rupture of blood vessels and a significant depletion in blood flow. Hypoxia-inducible factor 1 (HIF-1) is an oxygen-sensitive transcriptional activator that mediates cellular responses to low oxygen tension. Regarding the relationship with our chemokine of interest, the SDF-1, studies have reported an increase in the SDF-1 level as well as an upregulation of the HIF-1α level around the surgical site. This suggests that, during the initial stage of regeneration, hypoxic stress induced by injury plays a distinct role in contributing to the increase in SDF-1 expression, consequently leading to the selective homing and migration of CXCR4 + stem and progenitor cells to the ischemic injured area^13,14^. A similar hypoxia event can also be detected on the compression side of the PDL during OTM, where aseptic inflammation and obstruction of blood vessels occurred^22^. PDL expression of SDF-1, in accordance with the injury situation, can recruit distant BMSCs that comprise progenitor cells for the osteoblastic and hematopoietic lineage cells with the potential to develop into bone-resorbing osteoclasts^23,24^. Additionally, SDF-1 has the potential to promote periodontal tissue regeneration^25^. Considering these concepts, OTM, which involves PDL alteration, transient hypoxia from occlusion of PDL vessels, and aseptic inflammation, could also induce an increase in SDF-1 chemokine levels and subsequently lead to BMSC recruitment, activation, and proliferation.

Hatano et al^16^. tested the role of the SDF-1/CXCR4 axis in an M1 experimental tooth movement model using systemic injection of AMD3100, a CXCR-4 antagonist, in 6-week old rats. Compared to that in the control, the number of multinucleated osteoclasts in the PDL decreased after induced tooth movement on days 1 and 3, along with a significant reduction in the tooth movement rate from days 0 to 1. This suggests an impairment in osteoclastogenesis caused by the successful suppression of the SDF-1/CXCR4 axis^16^. More recently, a similar study comparing systemic and local serial injections of AMD3100 in an M1 OTM rat model concluded that local administration was equally effective in inhibiting the SDF-1/CXCR4 axis, with lower systemic side effects and better cost-effectiveness^17^. These supportive statements suggest the involvement of the SDF-1/CXCR4 axis in alveolar bone metabolism during OTM and successful localized inhibition using a receptor antagonist in this model. Further exploration into this chemokine involvement using a specific monoclonal neutralizing antibody targeting the SDF-1 chemokine itself is intriguing. In our study, unilateral tooth extraction of the maxillary M1, orthodontic force application to the maxillary M2, and localized injection of an anti-SDF-1 neutralizing monoclonal antibody were performed to examine the effects of blocking the SDF-1 chemokine on the RAP during OTM after adjacent tooth extraction. To identify the presence of SDF-1 gene expression and its location in this experimental model, and to observe the relationship between SDF-1 and the RAP in this model through localized injection of an anti-SDF-1 neutralizing antibody, the results were analyzed and separated into two parts.

**Part 1**. To confirm the presence of our targeted chemokine and its receptor in this experimental animal model, we observed a significant increase in SDF-1 expression on days 1, 3, and 7 on the experimental side after M1 extraction and M2 tooth movement, whereas a significant increase in CXCR4 expression was observed on day 7 (Fig. 2a,b). This confirmed an upregulated expression of SDF-1 mRNA in the M2 alveolar bone at the transcriptional level, the target in which the anti-SDF-1 monoclonal antibody was locally injected to neutralize this specific chemokine. Previous reports have also mentioned that SDF-1 expression is upregulated in the areas of fracture healing and ischemia^9,19^. Similar to bone fractures, healing of the extraction socket results in hematoma formation and exposure of the bone matrix and periosteum, leading to the expressions of inflammatory cytokines, chemokines, growth factors, angiogenic factors, and other small molecules. Recruitment of circulating progenitor cells and stem cell migrations occurred accordingly as normal biological processes. This chemotaxis of cells from nearby and distant sites to the injured area along chemical gradients was achieved via the upregulation of specific chemokines and chemoattracting cells presenting with their cognate receptors, including SDF-1 and its CXCR4 receptor^9^. In a stress fracture model in which repetitive loading of the ulna of a rat was performed to investigate bone fatigue injury and remodeling, the SDF-1 expression level was significantly increased and peaked at day 4 after the fracture was initiated^26^. Another study revealed increased SDF-1 mRNA expressions on days 2 and 3 in a mouse segmental live bone graft model, which is consistent with our results^27^.

Additionally, IF staining of SDF-1 revealed the presence and localization of this chemokine. Compared to the untreated control side, positive emissions were present at the PDL and adjacent bone marrow on the experimental side (Fig. 1a,b). Positive cells were most abundant on day 3, which coincides with a similar study^16^. This further validates the location and helps us decide both the injection period and location of the neutralizing SDF-1 monoclonal antibody and the observation period stated in Part 2 of this experimental design to optimally and cost-effectively block SDF-1 within specific time limits, where its expression would be the highest in our animal model.

**Part 2**. Experimental periods of 1, 3, and 7 days after OTM induction were used for the analysis based on previous studies demonstrating significant changes within these intervals^16,17^. Our results demonstrated a significant difference in the reduction of M2 tooth movement observed on day 3 in the experimental group compared to that in the two controls, measured using the reference line from the opposite M3 (Fig. 3b). Based on studies involving MIS and OTM to elicit the RAP, OTM was found to have a significant difference on day 3 when the number of osteoclasts was the highest^6,28^. TRAP and IHC staining of Cathepsin K supported this statement, where significantly fewer positively stained multinucleated osteoclasts were found on day 3 in the PDL of the M2 compression side on the SDF-1 antibody group (Figs. 5a,b, 6d,h). In addition, the systemic influence of a localized injection of the antibody was proven by enzyme-linked immunosorbent assay (ELISA) using peripheral blood serum, in which no significant differences in SDF-1 protein concentration were found among all groups at any time point (Supplementary Fig. 4). This demonstrated that confinement of the locally injected anti-SDF-1 antibody was accomplished, and any possible systemic effects related to the antibody injection were eliminated.

Moreover, IHC staining of IL-1β, IL6, and RANKL revealed significantly less positively stained areas on day 3 of the experimental group, suggesting that less inflammatory responses occurred at the region of interest (ROI) (Fig. 6a–c, e–g). This coincides with the frequency of anti-SDF-1 antibody injection, which was performed serially for only the first 3 consecutive days. Additionally, previous studies involving the blockage of this specific pathway also found significant differences in OTM as early as day 1^16,17^. However, in that study, on day 1, the initial OTM might have been caused solely by compression of the PDL once the appliance was applied to the tooth, which could explain why no significant difference was found among the three groups in our study. Interestingly, we noticed that M3 also had mesial tooth movement alongside M2, without having any appliance or force applied directly to the tooth. A significant difference was found on day 3, when less M3 tooth movement was measured, similar to the M2 mesial OTM (Fig. 3c). According to this result, the theory involving the presence of transseptal fibers embedded in the cementum and extending interproximally over the alveolar bone crest between two adjacent teeth could be a possible reason for consecutive M3 mesial tooth movement without any direct force application. With their ability to maintain mesiodistal contact and tooth position, several reports have mentioned that transseptal fibers could play key roles in tooth relapse after orthodontic treatment, especially in rotated teeth where they tend to relapse back into their original position^29–31^. During OTM, the PDL width on the distal side of the distal root increased at the alveolar crest level and decreased apically, consistent with mesial tipping tooth movement^30^. In correlation with the increased PDL space from the orthodontic force that led to the stretching of transseptal fibers, this could consequently result in the engagement of the adjacent smaller and isolated M3 to move mesially along with M2. However, these movements are only assumed to occur, and more research is needed to prove this hypothesis.

Although not statistically significant, there is a notable trend of reduction in BV/TV and Tb.Th in all groups further along in the experimental period from day 1 to 7 (Supplementary Fig. 3). An interpretation of these results is that the animal model of M1 tooth extraction and M2 tooth movement elicits RAP, leading to an active catabolic bone resorption response that results in a reduction in bone volume over the course of tooth movement, similar to that in previous studies involving MIS or with OTM experimental designs^6,32,33^. However, herein, with anti-SDF-1 neutralizing monoclonal antibody injection in the experimental group, significantly higher bone mineralization was found in the experimental group on day 3, as shown by the statistically increased BMD values.

Our study demonstrated a significant reduction in SDF-1 and CXCR4 gene expressions on day 3 in the experimental group following chemokine neutralization by serial local injections of an anti-SDF-1 antibody, confirming the successful inhibition of the targeted axis (Fig. 7a,b). This appears together with a significant reduction in the IL-1β, RANKL, and OPG genes on day 3 in the experimental group (Fig. 7c–e). Additionally, IF data confirmed a significant reduction in SDF-1 expression on the compression side of the M2 mesial root on day 3 in the experimental group compared to the two control groups (Fig. 4a,b). In agreement with studies where OTM was combined with MIS procedures, eliciting the RAP showed increased levels of osteoclastogenic activity around day 3 and up to day 7^6,34^, which supports our results, where significantly different results on inflammatory and osteoclastogenesis-related factors could be observed on day 3, but not on day 1. Corresponding to our experimental design, where local injections were performed only for the first 3 days, the inhibitory effects of the locally injected antibody were temporary, and its termination resulted in a resumption of normal inflammation and the bone remodeling process as demonstrated on day 7 of all tested factors, where no significant differences were observed between the controls and experimental groups.

Interestingly, our experiment demonstrated a significant reduction in OPG expression on days 1 and 3 of the experimental group (Fig. 7e). This result is consistent with a previous report showing that OPG expression increased on day 14 as a result of SDF-1/CXCR4 blockage via AMD-3100 in an OTM model^17^. Focusing more on inflammation than on bone healing, the discrepancy in the OPG expression results could be related to the time point at which the OPG mRNA level was measured and the design of the experiment. Another possible explanation for this phenomenon is that directly blocking the SDF-1 chemokine using an antibody is different from blocking its receptor using the CXCR4 antagonist AMD-3100. With SDF-1 functioning as a master regulator of stem and progenitor cell migration^9,27^, diminishing this chemokine could lead to less migration of the mesenchymal stem cells (MSCs) homing to the injured area, resulting in fewer MSCs differentiating into pre-osteoblast precursor cells, resulting in lower OPG levels in our selected ROI. An in vitro study investigating the angiogenic process modulation by OPG found that the expression of OPG mRNA markedly increased six-fold after 4 h, and three-fold after 24 h of stimulation with SDF-1. This study suggests that OPG expression is modulated by SDF-1 as early as 4 h, which potentially explains the significant reduction in OPG expression as early as 1 day after SDF-1 neutralization in the experimental group^35^.

Further supporting our assumption, Kitaori et al^27^. showed that SDF-1 promotes the migration of MSCs in vitro in a dose-dependent manner and that blocking treatment significantly reduced the number of migrated cells. Regarding its function in MSC recruitment for proper bone repair, blocking it using a neutralizing SDF-1 antibody or CXCR4 antagonist inhibits cell migration, resulting in significantly decreased new bone formation^27^. Toupadakis et al^36^. also investigated the role of SDF-1/CXCR4 signaling in a mouse bone fracture model in which long-term AMD3100 was administered. Impairment of the homing and differentiation of stem cells to the injured site resulted in significantly reduced cartilage volume, callus size, and bone mineralization, with significant reductions in the expressions of several mRNA involved in the mineralization of cartilage and bone and revascularization. Additionally, mRNA levels, which play a role in MSC proliferation, osteoblast differentiation, and osteoclast survival, were also significantly decreased^36^. This summarizes that the disruption of this signaling pathway significantly affects the bone healing, suggesting that short-term blockage of SDF-1 could alleviate the inflammatory and bone resorption processes but would interfere with bone healing if continuous administration of the antibody continues.

## Conclusion

We found that evoking the RAP by M1 tooth extraction and initiating M2 OTM significantly increased the expression levels of SDF-1 chemokines in the SDF-1/CXCL4 axis, both histologically and genetically. Consequently, blocking these targeted SDF-1 chemokines using serial local injection of anti-SDF-1 neutralizing monoclonal antibody showed a significant reduction of OTM on day 3 of the experiment, supported by histological and relative gene expression results indicating less osteoclast accumulation and fewer inflammatory responses.

### Supplementary Information

Supplementary Figures.

## Data Availability

The datasets generated and/or analyzed in this study are available from the corresponding author upon reasonable request.

## Abbreviations

M1: Maxillary first molar
M2: Maxillary second molar
OTM: Orthodontic tooth movement
RAP: Regional acceleratory phenomenon
SDF-1: Stromal cell-derived factor 1
