# Author Correction: SDF-1 involvement in orthodontic tooth movement after tooth extraction

**DOI:** 10.1038/s41598-024-56881-x

**Published:** 2024-03-14

**Authors:** Duangtawan Rintanalert, Yuji Ishida, Albert Chun-shuo Huang, Kasumi Hatano-sato, Kai Li, Pintu-on Chantarawaratit, Risa Usumi-fujita, Jun Hosomichi, Takashi Ono

**Affiliations:** 1 Department of Orthodontic Science, Graduate School of Medical and Dental Sciences, Tokyo Medical and Dental University (TMDU), Yushima 1-5-45, Bunkyo-ku, Tokyo, 113-8510 Japan; 2 Department of Orthodontics, Faculty of Dentistry, Chulalongkorn University, Bangkok, 10330 Thailand

Correction to: *Scientific Reports* 10.1038/s41598-024-55632-2, published online 29 February 2024

The original version of this Article contained an error in the name of the author Albert Chun-shuo Huang, which was incorrectly given as Chun-shuo Huang.

The original Article and accompanying Supplementary Figures file have been corrected.

