# Supplementary material for: SDF-1 involvement in orthodontic tooth movement after tooth extraction
Source: Sci Rep. 2024 Feb 29;14:5048. doi: 10.1038/s41598-024-55632-2 (PMC10904391; doi:10.1038/s41598-024-55632-2)
Supplement: Supplementary file 1 — Supplementary Figures. [file 41598_2024_55632_MOESM1_ESM.pdf]

SDF-1 Involvement in Orthodontic Tooth Movement After Tooth Extraction

Duangtawan Rintanalert[1, 2], Yuji Ishida[1], Albert chun-shuo HUANG[1], Kasumi Hatano-sato[1], Kai Li[1], Pintu-on Chantarawaratit[2], Risa Usumi-fujita[1], Jun Hosomichi[1], Takashi Ono[1]

[1] Department of Orthodontic Science, Graduate school of Medical and Dental Sciences, Tokyo Medical and Dental University (TMDU), Tokyo 113-8510, Japan

[2] Department of Orthodontics, Faculty of Dentistry, Chulalongkorn University, Bangkok 10330, Thailand

* Corresponding author at: Department of Orthodontic Science, Tokyo Medical and Dental University, 113-8549, Yushima 1-5-45, Bunkyo-ku, Tokyo, Japan

Email address: yjis.orts@tmd.ac.jp

Keywords: Orthodontic Tooth Movement (OTM), Stromal Cell-Derived Factor 1 (SDF-1), Tooth Extraction, Regional Acceleratory Phenomenon (RAP), Neutralizing Antibody

Abbreviations: Maxillary first molar; M1, Maxillary second molar; M2, Orthodontic Tooth Movement; OTM, Regional Acceleratory Phenomenon; RAP, Stromal Cell-Derived Factor 1; SDF-1

Supplementary Material

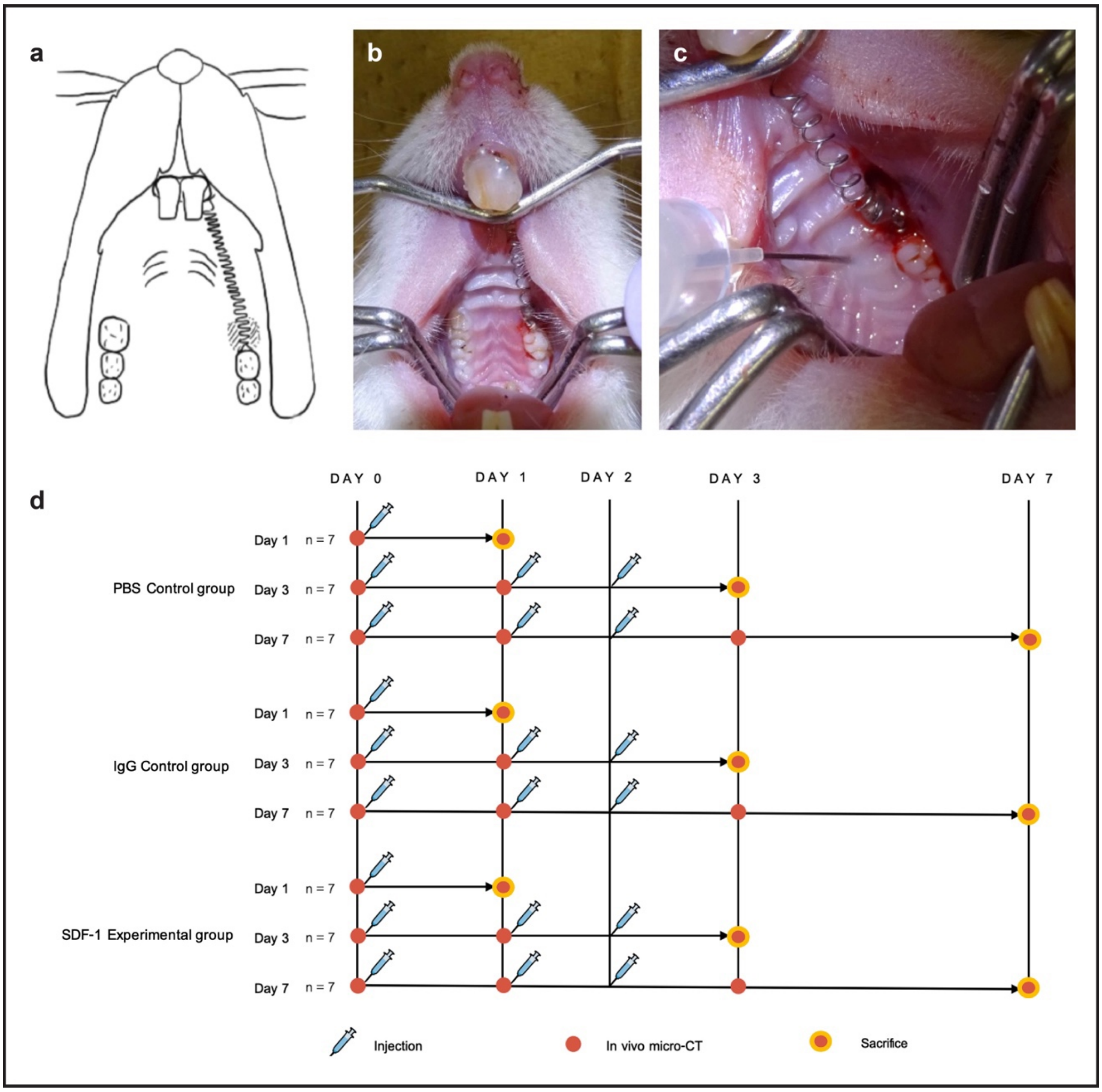

Supplementary Figure 1. M1 extraction and M2 OTM surgical model (**a**), intraoral appliance in rat OTM model (**b**), and injection site at palatal mucosa (**c**). Diagram of the experimental design – injections, in vivo micro-CT frequency, and the day of sacrifice (**d**).

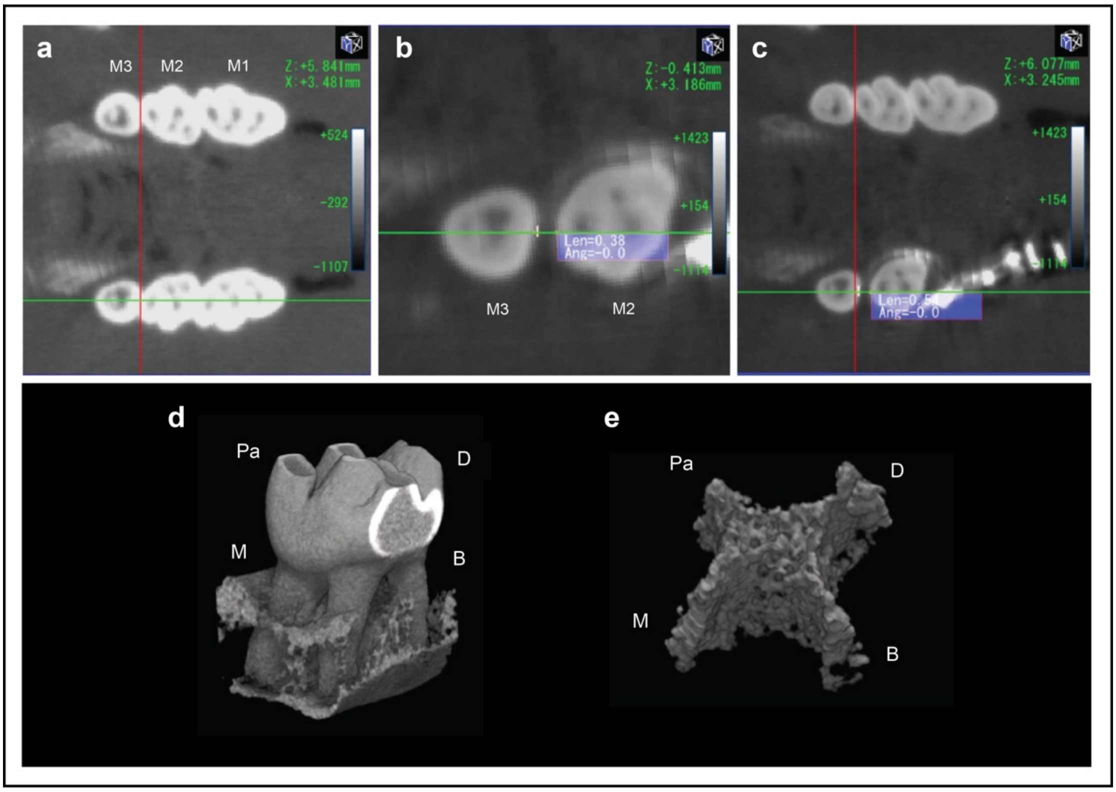

Supplementary Figure 2. Pre-operative micro-CT data for tooth movement measurement baseline (**a**). Tooth movement measurements from the mesial surface of M3 (**b**) and the red reference line (**c**). LT, left; RT, right; M, mesial; D, distal; M1, the maxillary first molar; M2, the maxillary second molar; M3, the maxillary third molar. Interradicular alveolar bone of the maxillary M2 (**d**). Region of interest for the structural morphometry analysis (**e**). M, mesial; D, distal; Pa, palatal; B, buccal.

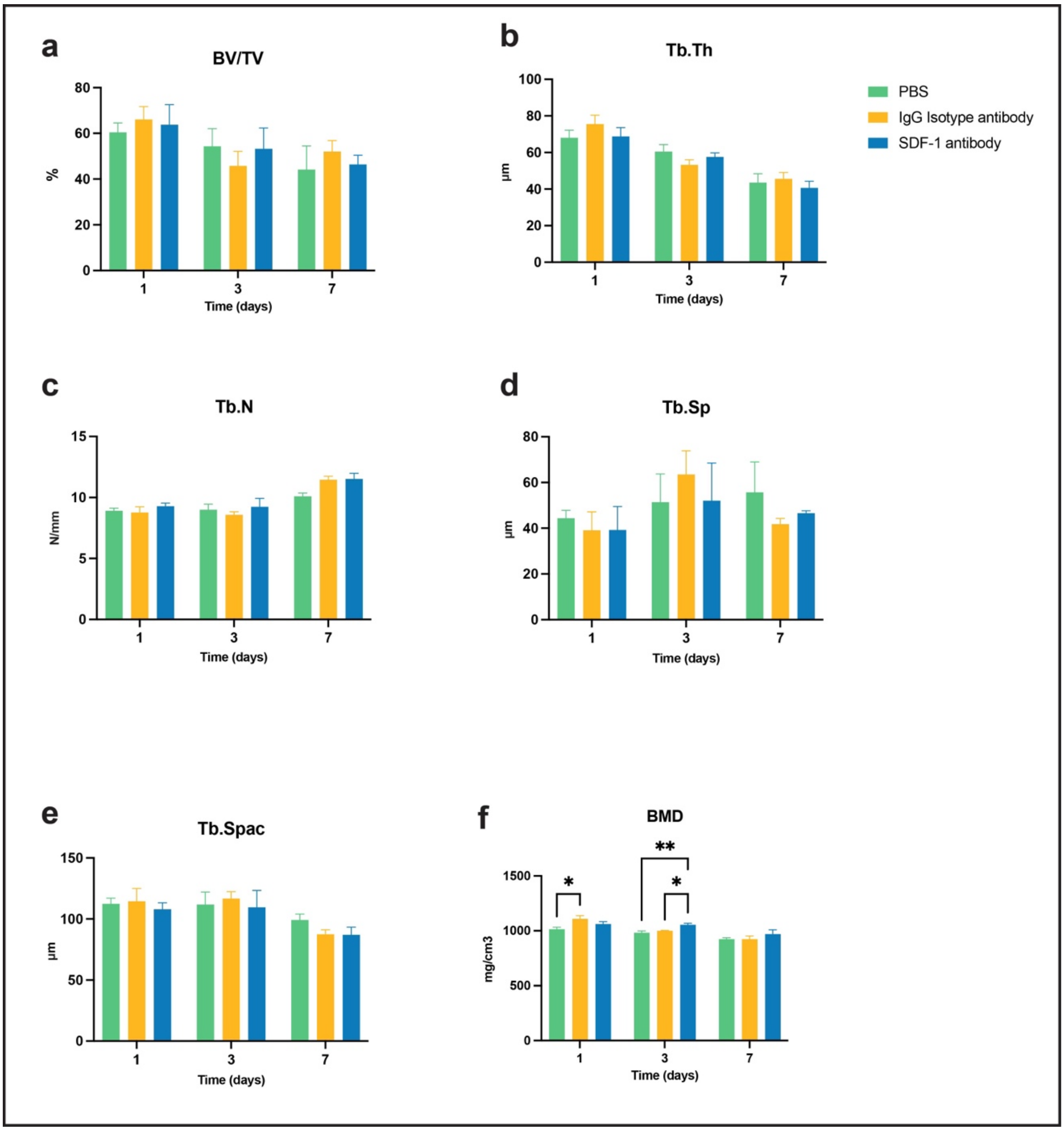

Supplementary Figure 3. Micro-CT results of the measured parameters including: (**a**) bone volume fraction (BV/TV); (**b**) trabecular thickness (Tb.Th); (**c**) trabecular number (Tb.N); (**d**) trabecular separation (Tb.Sp); (**e**) trabecular spacing (Tb.Spac); and (**f**) bone mineral density (BMD). Structural morphometry analyses were performed at the selected region of interest being the interradicular alveolar bone of the maxillary M2. Results indicate a significant increase in BMD value on day 3 in the experimental group compared to that in the two control groups. Values are presented as mean ± standard deviation (n = 3). * $p < 0.05$, ** $p < 0.01$

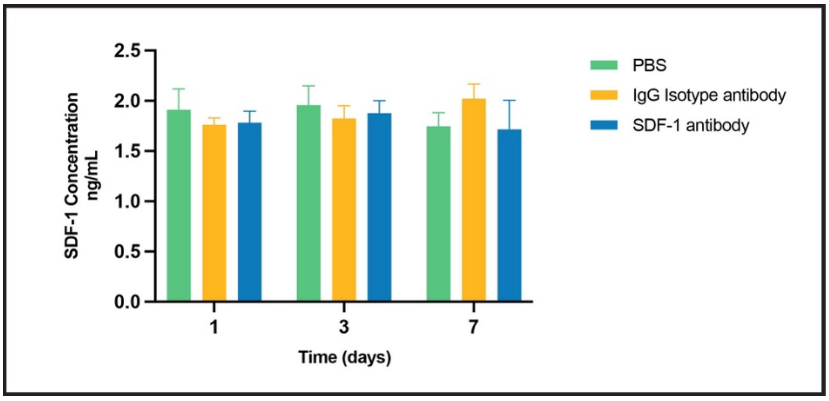

Supplementary Figure 4. Systemic concentration of SDF-1 protein in the peripheral blood serum on days 1, 3, and 7. Values are presented as means ± standard deviation (*n* = 7).
